# Emergence and Spread of Different ESBL-Producing *Salmonella enterica* Serovars in Hospitalized Horses Sharing a Highly Transferable IncM2 CTX-M-3-Encoding Plasmid

**DOI:** 10.3389/fmicb.2020.616032

**Published:** 2020-12-17

**Authors:** Ziv Dor, Anat Shnaiderman-Torban, Kira Kondratyeva, Maya Davidovich-Cohen, Assaf Rokney, Amir Steinman, Shiri Navon-Venezia

**Affiliations:** ^1^Department of Molecular Biology, Ariel University, Ariel, Israel; ^2^Koret School of Veterinary Medicine, The Robert H. Smith Faculty of Agriculture, Food and Environment, The Hebrew University of Jerusalem, Rehovot, Israel; ^3^Government Central Laboratories, Ministry of Health, Jerusalem, Israel; ^4^The Dr. Miriam and Sheldon G. Adelson School of Medicine, Ariel University, Ariel, Israel

**Keywords:** *Salmonella enterica*, WGS, ESBL, serovars, IncM2, *bla*_*CTX–M–3*_, MDR plasmid, horizontal transfer

## Abstract

*Salmonella enterica* is a major causative pathogen of human and animal gastroenteritis. Antibiotic resistant strains have emerged due to the production of extended-spectrum β-lactamases (ESBLs) posing a major health concern. With the increasing reports on ESBL-producing Enterobacterales that colonize companion animals, we aimed to investigate ESBL dissemination among ESBL-producing *Salmonella enterica* (ESBL-S) in hospitalized horses. We prospectively collected ESBL-S isolates from hospitalized horses in a Veterinary-Teaching Hospital during Dec 2015–Dec 2017. Selection criteria for ESBL-S were white colonies on CHROMagarESBL plates and an ESBL phenotypic confirmation. *Salmonella enterica* serovars were determined using the Kaufmann-White-Le-Minor serological scheme. ESBL-encoding plasmids were purified, transformed and compared using restriction fragment length polymorphism (RFLP). Whole genome sequencing (Illumina and MinION platforms) were performed for detailed phylogenetic and plasmid analyses. Twelve ESBL-S were included in this study. Molecular investigation and Sequence Read Archive (SRA) meta-analysis revealed the presence of three unique *Salmonella enterica* serovars, Cerro, Havana and Liverpool, all reported for the first time in horses. PFGE revealed the clonal spread of *S.* Cerro between seven horses. All twelve isolates carried *bla*_*CTX–M–*__3_ and showed an identical multidrug resistance profile with co-resistance to trimethoprim/sulfamethoxazole and to aminoglycosides. Plasmid RFLP proved the inter-serovar horizontal spread of a single *bla*_*CTX–M–*__3_-encoding plasmid. Complete sequence of a representative plasmid (*S*. Havana strain 373.3.1), designated pSEIL-3 was a -86.4 Kb IncM2 plasmid, that encoded nine antibiotic resistance genes. pSEIL-3 was virtually identical to pCTX-M3 from *Citrobacter freundii*, and showed high identity (>95%) to six other *bla*_*CTX–M–*__3_ or *bla*_*NDM–*__1_ IncM2 broad host range plasmids from various Enterobacterales of human origin. Using a specific six gene-based multiplex PCR, we detected pSEIL-3 in various Enterobacterales species that co-colonized the horses’ gut. Together, our findings show the alarming emergence of ESBL-S in hospitalized horses associated with gut shedding and foal morbidity and mortality. We demonstrated the dissemination of CTX-M-3 ESBL among different *Salmonella enterica* serovars due to transmission of a broad host range plasmid. This report highlights horses as a zoonotic reservoir for ESBL-S, including highly transmissible plasmids that may represent a ‘One-Health’ hazard. This risk calls for the implementation of infection control measures to monitor and control the spread of ESBL-S in hospitalized horses.

## Introduction

*Salmonella enterica* is the major causative pathogen of human and animal Salmonellosis (Scallan et al., 2011; Omer et al., 2018). Human Salmonellosis recently poses a major health concern due to the dissemination of multidrug resistant (MDR) strains that produce extended-spectrum β-lactamases (ESBLs) that limit the appropriate treatment options (Antonelli et al., 2019; Jajere, 2019). ESBL-producing *Salmonella enterica* (ESBL-S) are increasingly reported from livestock animals (European Centre for Disease Prevention and Control., 2019). Shared ESBL-S serovars among livestock and humans suggest that food animals are possible zoonotic reservoir for this human-associated pathogen (Sjölund-Karlsson et al., 2013).

In the last decade, along with food animals, ESBL-producing Enterobacterales colonization in companion animals is steadily increasing (Doi et al., 2017). Although the zoonotic potential of these bacteria is still enigmatic, there is a consensus regarding their role as being a reservoir for antibiotic resistance, and as a possible hazard to human health due to the close physical contact between companion animals and humans (Madec et al., 2017).

Horses in specific are in close interaction with humans and children in various interfaces including private use, sport events and as therapeutic animals. As such, they may serve as a zoonotic source for antibiotic resistant pathogens. Horses have been shown previously to be colonized and infected with various clinically important pathogens including methicillin-resistant *Staphylococcus aureus* (MRSA) (Tirosh-Levy et al., 2015), *Acinetobacter baumannii*, and various ESBL-producing enteric pathogens (Walther et al., 2018; Shnaiderman-Torban et al., 2019). As for the genus *Salmonella*, horses may be sub-clinically infected with the bacterium or suffer from clinical signs which may vary from mild disease as fever and dehydration, to diarrhea, colic and manifestations of septicemia (Hernandez et al., 2014; Cummings et al., 2016). However, reports on ESBL-S strains in horses are still rare. A report from Germany described an SHV-12-producing *S*. Newport causing an outbreak in an equine hospital, which led to a three-month facility closure (Rankin et al., 2005). Another report from the United States described 11 ESBL-S clinical isolates from an equine referral hospital that belonged to various serovars including Braenderup, Anatum, Agona, Rubislaw, and Newport (Leon et al., 2018).

In a previous study, we investigated the shedding rate of ESBL-producing Enterobacterales in farm horses versus hospitalized horses and observed a significant increase in ESBL shedding rate among hospitalized horses together with first isolation of three colonizing isolates that we identified as ESBL-S isolates (Shnaiderman-Torban et al., 2020). The present study investigated and characterized the molecular epidemiology of ESBL-S isolates that were isolated during our surveys, together with ESBL-S isolates recovered from clinical infections from hospitalized horses during the study period. We aimed to describe the emergence of ESBL-S in hospitalized horses and to explore the dissemination of ESBL in this important pathogen.

## Materials and Methods

### Isolation of ESBL-Producing Enterobacterales From Hospitalized Horses

During a prospective surveillance study of ESBL-producing Enterobacterales (ESBL-E) gut colonization in hospitalized horses that we performed in the Koret School of Veterinary Medicine-Veterinary Teaching Hospital (KSVM-VTH) in Israel (Dec 2015–Dec 2017), rectal swabs were collected from horses on admission and after 72 h of hospitalization. The study protocol was approved by the Internal Research Review Institution Committee (Protocol number: KSVM-VTH/15_2015). Isolation of ESBL-E from swabs was performed after swab enrichment in Tryptic Soy Broth supplemented with Ampicillin (100 mg/L), and an overnight incubation at 37°C to increase sensitivity of detection (Jazmati et al., 2016). After incubation, samples were plated onto CHROMagarESBL plates (HyLabs, Rehovot, Israel). In addition, *Salmonella enterica* clinical isolates recovered from horses during the study period that were processed at the Clinical Microbiology Lab at the Meir Medical Center, Kfar Saba, Israel, were collected and stored for retrospective molecular characterization.

### Isolation of ESBL-Producing *Salmonella enterica* and the Identification of ESBL Genes

Following the former described procedure, all the white colonies that were obtained on the CHROMagarESBL plates, suspected as ESBL-producing *Salmonella enterica* (ESBL-S) were further isolated onto selective *Salmonella/Shigella*-agar plates (HyLabs) following verification using the slide agglutination polyvalent serum assay (Remel Inc., United States). All ESBL-S isolates (both fecal and clinical isolates) were identified by the VITEK 2 automated system (Biomerieux, United States) together with antibiotic susceptibility testing using AST-N270 and AST-GN65 cards. Susceptibility results were interpreted according to the Clinical and Laboratory Standards Institute (CLSI) guidelines. All isolates were confirmed for a positive ESBL production phenotype using the cephalosporin/clavulanic-acid combination disk assay (Oxoid, United Kingdom). The *bla*_*CTX–M*_ genes were identified by multiplex-PCR (Woodford et al., 2006) and Sanger sequencing (Macrogen, Netherlands). Sequences were analyzed (Snap-Gene) and compared with NCBI database to identify the specific ESBL gene allele.

### *Salmonella enterica* Serovar Identification and Pulsed Field Gel Electrophoresis (PFGE)

*Salmonella enterica* serotyping was performed using the Kauffmann-White-Le Minor scheme (Le Minor et al., 1982). *Xba*I-restricted (New England BioLabs) PFGE was performed according to the PulseNet International Standard Protocol (Ribot et al., 2006) with *S.* Braenderup H9812 as a reference strain. The PFGE fingerprinting patterns were analyzed with BioNumerics software (version 7.6.3, Applied Maths, Sint-Martens-Latem, Belgium). The unweighted-pair group method using average linkages (UPGMA) clustering method and Dice similarity coefficients were used (1% optimization and 1% tolerance). Isolates were defined as genetically related if they presented ≥ 98% PFGE similarity.

### Meta Data of the NCBI Sequence Read Archive (SRA) for Statistical Analysis

We explored the global occurrences and the isolation sources of the *Salmonella enterica* serovars using the NCBI SRA data. In order to perform the mata-analysis, we retrieved the SRA accession numbers and meta-data for all the publically available isolates using the NCBI E-Utilities. Statistical correlations between the serovar type and the isolation source were calculated using Phi coefficients with *p*-values < 0.01. *P*-values were corrected for multiple tests in step-down method using Bonferroni adjustments (alpha = 0.01). All statistical analyses were performed using Python statistics modules.

### *Salmonella enterica* Whole Genome Sequencing (WGS) and Data Analysis

Total DNA was isolated using Blood and Tissue kit (Qiagen, Hilden, Germany) according to the manufacturer’s protocol. WGS was performed by Illumina MiSeq platform using 2 × 250 paired-end libraries prepared with the NEBNext Ultra II FS DNA Library Prep Kit. Assembly was performed using SPAdes-3.11.1. Plasmid replicon types and antibiotic resistance genes (ARGs) were identified using the Center for Genomic Epidemiology (CGE) pipeline.

### Whole Genome Multi Locus Sequence Typing (wgMLST) Phylogenetic Analysis

The EnteroBase database was searched for sequences predicted as *S*. Cerro, *S*. Havana, or *S*. Liverpool, according to the SISTR1 and SeqSero2 algorithms. Strains with source country metadata were selected for a phylogenetic analysis and for comparison with the Israeli sequences. A GrapeTree depiction of a NINJA NJ tree based on the wgMLST allelic distances was generated for each serovar population.

### Purification and Characterization of *Salmonella enterica* ESBL-Encoding Plasmids

ESBL-encoding plasmid DNA was extracted using the Plasmid Midi Kit (Qiagen) following the manufacturer’s instructions. Plasmids were transformed into electro-competent *Escherichia coli* DH10B and transformants were selected on ampicillin containing LB plates (100 mg/L), followed by *bla*_*CTX–M*_ PCR screening (Woodford et al., 2006). A second transformation and plasmid purification was performed to ensure plasmid purity. ESBL-encoding plasmids purified from all the 12 ESBL-S isolates were compared using RFLP analysis following restriction with *Sac*I, *Eco*RI and *Hin*dIII (New England BioLabs) and electrophoresis.

### Complete Sequencing and Annotation of *bla*_*CTX–M–*__3_-Encoding Plasmid pSEIL-3

Since ten of 12 isolates harbored CTX-M-3-encoding plasmids with identical RFLP patterns, one representative plasmid (pSEIL-3 from *S*. Havana strain 373.3.1) was sequenced using MinION device (Oxford Nanopore Technologies, ONT, Oxford, United Kingdom) following hybrid assembly, resulting in a complete plasmid sequence (Wick et al., 2017). Plasmid DNA (200 ng) fragment library was prepared (SQK-RBK004 ONT Rapid barcoding sequencing kit) according to the manufacturer’s instructions, and loaded onto the MinION flow cell FLO-MIN106. The hybrid read set (WGS Illumina and Nanopore reads) was assembled using Unicycler (v0.4.0) to yield a single circular plasmid designated pSEIL-3, annotated by RAST (Aziz et al., 2008). Replicon type assignment, ARG content, virulence genes and IS elements identification were performed using the CGE pipeline, and by ISfinder (Siguier et al., 2012). Homologous plasmids were identified from the NCBI Nucleotide (nt/nr) database using BLASTn search. Linear plasmid maps were generated using Easyfig-2.2.3.

### GenBank Submission

WGS Illumina reads of the three *S.* serovars were deposited in the NCBI Sequence Read Archive database under project number PRJNA559324 (Table 2). The complete pSEIL-3 sequence isolated from *S.* Havana strain 373.3.1 (BioSample SAMN12532154) was submitted to the NCBI Nucleotide database under the accession number MN380440.

### Conjugation Experiments of pSEIL-3

Conjugation experiments were performed with *S.* Cerro strain 339.3.3 and *S.* Havana strain 373.3.1 as the donor strains and *Klebsiella pneumoniae* B199 (resistant to nalidixic-acid) and *E. coli* J53 (rifampicin resistant) as the recipient strains. Filter-mating was performed (donor and recipient, 1:1 ratio) on LB plates followed by selection of transconjugant colonies on LB agar plates containing ceftriaxone (2 mg/L) and either nalidixic acid (64 mg/L) or rifampicin (300 mg/L). Transconjugants were verified by the colony color obtained on CHROMagarESBL plates and by PCR detection of *bla*_*CTX–M–*__3_, and then were subjected to the VITEK 2 for antibiotic susceptibility testing.

### Molecular Screening for the Presence of pSEIL-3

A novel six-gene multiplex PCR scheme for the molecular screening of pSEIL-3 was developed. The primers were designed (Table 3) based on the sequences of six genes whose combination was unique according to the NCBI Nucleotide database search. The multiplex PCR was performed with PCRBIO HS Taq Mix Red (PCRBIO-systems, United Kingdom) at the following conditions: denaturation at 95°C for one minute, 29 cycles of denaturation (95°C, 15 s), annealing (61.1°C, 15 s) and elongation (72°C, 90 s).

## Results

### ESBL-Producing *Salmonella enterica* Isolates Recovered From Hospitalized Horses

Overall, 12 ESBL-S isolates were recovered from 12 hospitalized horses during the study period. All these strains were isolated > 72 h after admission and therefore were defined as nosocomial (Table 1). Ten out of the 12 horses were also sampled on admission, as part of an ESBL surveillance study, and two of them were found to be colonized with an ESBL-producing Enterobacterales (ESBL-E) strain. Nine out of the 12 horses (75%) that were colonized with an ESBL-S were also found to be colonized with different ESBL-E species. Diverse ESBL-E colonizing species were found: *E. coli* (*n* = 7), *K. pneumoniae* (*n* = 4), *Klebsiella oxytoca* (*n* = 2), and *Citrobacter freundii* (*n* = 1) (Table 1). Ten of the ESBL-S isolates originated from rectal swabs, of which, four were sampled from asymptomatic horses, and six from horses with clinical signs of gastroenteritis. Two additional ESBL-S isolates were clinical isolates that caused joint and umbilicus infections. The majority of the horses from which ESBL-S was recovered were neonates (8/12, 67%, Table 1), which were all diagnosed with sepsis (Wong et al., 2018), presenting various clinical signs. These foals were all treated with ampicillin and amikacin, and if they suffered from diarrhea, metronidazole therapy was added. Five out of eight (62.5%) died or were euthanized during hospitalization.

**TABLE 1:** Molecular and epidemiological characteristics of the 12 ESBL-producing *Salmonella enterica* (ESBL-S) isolates included in this study and their equine host.

**Isolate**	**ESBL-producing Enterobacterales carriage status on admission**	**ESBL-S Isolation date**	**Equine host**	**Colonization or infection (outcome)^*a*^**	***Salmonella* serovar**	***bla*_*CTX–M–*__3_- plasmid^*c*^**	**Co-colonizing ESBL-E^*d*^**
72.2.3	Negative	20 Dec 2015	Mare	Gut colonization (S)	Havana (group G)	pSEIL-3-like IncM2	*Citrobacter freundii*
124.2.3	Negative	28 Jan 2016	Gelding	Gut colonization (S)	Havana (group G)	pSEIL-3-like IncM2	*E. coli*
229.2.2	Negative	12 Mar 2016	Foal	Gastroenteritis^*b*^ (S)	Cerro (group K)	pSEIL-3-variant^*e*^	*E. coli*^*e*^
302.2.1	Negative	18 Apr 2016	Mare	Gut colonization (S)	Cerro (group K)	pSEIL-3-like IncM2	*E. coli K. oxytoca*^*f*^
320.2.3	Positive (ESBL-K. *pneumoniae*)	30 Apr 2016	Foal	Gastroenteritis^*b*^ (D)	Cerro (group K)	pSEIL-3-like IncM2	*E. coli*^*f*^ *K. pneumoniae*
322.2.2	Negative	5 May 2016	Foal	Gastroenteritis^*b*^ (D)	Cerro (group K)	pSEIL-3-like IncM2	*E. coli*^*f*^ *K. pneumoniae*^*f*^
303.4.3	Negative	9 May 2016	Foal	Gastroenteritis (D)	Cerro (group K)	pSEIL-3-like IncM2	*E. coli*^*f*^ *K. oxytoca*^*f*^
339.3.3*	Negative	May 2016	Foal	Umbilical infection (D)	Cerro (group K)	pSEIL-3	*E. coli*^*f*^ *K. pneumoniae*^*f*^
347.2.2	Positive (ESBL-*E. coli*)	30 May 2018	Mare	Gut colonization (S)	Cerro (group K)	pSEIL-3-varient^*d*^	*K. pneumoniae*^*f*^
373.3.1*	Negative	Nov 2016	Foal	Infected joint (S)	Havana (group G)	pSEIL-3	Unknown
667220	Unknown	21 Dec 2017	Foal	Gastroenteritis^*b*^ (S)	Liverpool (group E4)	pSEIL-3-like IncM2	Unknown
667275*	Unknown	31 Dec 2017	Foal	Gastroenteritis^*b*^ (D)	Liverpool (group E4)	pSEIL-3	Unknown

^*a*^Outcome status ‘S’ - survival; ‘D’ - death. ^*b*^ESBL-S was recovered from diarrhea specimen. ^*c*^The non-sequenced *bla*_*CTX–M–*__3_-encoding plasmids that possessed the same RFLP pattern were designated pSEIL-3-like IncM2 plasmids. ^*d*^ESBL-producing Enterobacterales isolates that co-colonized the same horse and were recovered at the same sampling time together with the ESBL-S. All were PCR-positive for *bla*_*CTX–M–*__1_ –group (Figure 1). ^*e*^pSIEL-3-like plasmids are *bla*_*CTX–M–*__3_-encoding plasmids that showed a different RFLP pattern compared to pSEIL-3 but were positive in the pSIEL-3-specific multiplex PCR. ^*f*^Non-Salmonella ESBL-producing Enterobacterales isolates that co-colonized the horses gut together with ESBL-S and were found to carry pSEIL-3 by the pSEIL-3-specific multiplex PCR. *Salmonella enterica isolates sent to WGS; Unknown – The horse was not screened for ESBL-E carriage during hospitalization due to a positive ESBL-S clinical culture.

**FIGURE 1:**
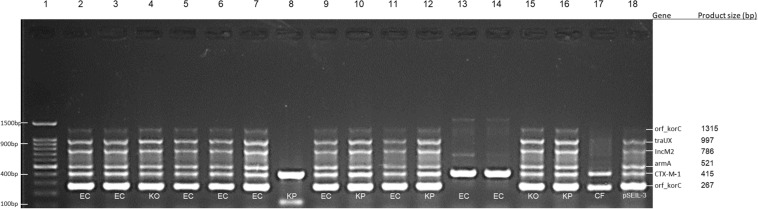
Multiplex PCR for screening of pSEIL-3 in non-Salmonella CTX-M-1-producing Enterobacteriales species co-colonizing eight horses. Six-gene-multiplex PCR amplification for the detection of pSIEL-3 was performed on 16 CTX-M-1 positive ESBL-E isolates co-colonizing (together with ESBL-S) eight horses. foal 229, lane 2; foal 303, lanes 3–6; foal 320, lanes 7–8; foal 322, lanes 9–10; foal 339, lanes 11–12; gelding 124, lane 13; mare 302, lanes 14–15; mare 347, lane 16; mare 72, lane 17; pSIEL-3, lane 18; DNA 100 bp ladder, lane 1. EC - E. coli; KP - K. pneumoniae; KO - K. oxytoca; CF - Citrobacter freundii.

### Serovars, Genotyping and Antibiotic Susceptibility Profiles

ESBL-S isolates belonged to three different serovars – Cerro (*n* = 7), Havana (*n* = 3), and Liverpool (*n* = 2), with Cerro being the major serovar, representing more than 50% of the isolates (Table 1). All the seven ESBL-producing *S.* Cerro isolates clustered in time (a two-month period) and PFGE genotyping suggested the clonal expansion of this serovar (87.8–100% isolate identity, Supplementary Figure 1).

All the 12 ESBL-S isolates carried *bla*_*CTX–M–*__3_ and showed an identical MDR profile independent with their serovar antibiotic susceptibility testing showed resistance to ceftriaxone, aminoglycosides, amikacin, tobramycin, gentamicin, and trimethoprim-sulfamethoxazole. Isolates were susceptible to carbapenems, quinolones and fosfomycin (Supplementary Table 1).

### WGS of ESBL-S Serovars and Identification of Plasmid Replicons and Resistome

To further explore the three ESBL-S serovars identified in the equine population, we performed WGS of three representative isolates, one of each serovar (data was deposited under project number PRJNA559324 in the GenBank). The WGS data is summarized in Table 2. Sequence types were identified *in silico*, and plasmid replicon analysis revealed that all three serovars harbored common IncM2 and ColRNAI plasmids, accompanied by other plasmids, that were unique for each isolate. Alongside with *bla*_*CTX–M–*__3_, they all encoded a wide resistome encompassing nine to 12 ARGs that correlated with their susceptibility profiles (Table 2).

**TABLE 2:** Description of WGS data of three equine ESBL-producing *Salmonella enterica* serovars.

***Salmonella enterica* strain (Bio Sample No).**	**Serovar/ST^*a*^**	**Genome size/GC%**	**N50 bp/L50**	**No. of ORFs/RNA/ARGs**	**Plasmid replicon^*b*^**	**Plasmids resistome^*c*^**	**Resistance pattern^*d*^**
339.3.3 (SAMN12532153)	Cerro ST1593	4.76 Mb/52.22	17575/8	4820/88/12	IncM2	*aac(3)-Iid*-like, *aadA2*, *armA*, *blaCTX-M-3*, *blaTEM-1B*, *dfrA12*, *mph(E)*-like, *msr(E)*, *sul1*, *sul2*	CTX, AMC(I), AMK; GEN; TOB, TMS
					IncI1	*aadA1*, *dfrA1*	
					ColRNAI	None	
373.3.1 (SAMN12532154)	Havana ST5248	4.77 Mb/52.16	407943/4	4836/96/9	IncM2	*aac(3)-Iid*-like, *aadA2*, *armA*, *blaCTX-M-3*, *blaTEM-1B*, *dfrA12*, *mph(E)*-like, *msr(E)*, *sul1*, *sul2*	CTX, AMC(I), AMK; GEN; TOB, TMS
					Col156	None	
					ColRNAI	None	
667275 (SAMN12532152)	Liverpool ST1959	4.9 Mb/52.15	762498/3	5007/100/12	IncM2	*aac(3)-Iid*-like, *aadA2*, *armA*, *blaCTX-M-3*, *blaTEM-1B*, *dfrA12*, *mph(E)*-like, *msr(E)*, *sul1*, *sul2*	CTX, AMC(I), AMK; GEN; TOB, TMS; CIP(I)
					IncX2	*qnrS1*, *tet(A)*-like	
					ColRNAI	None	

*^*a*^PubMLST (https://pubmlst.org/salmonella). ^*b*^PlasmidFinder 2.1 (https://cge.cbs.dtu.dk/services/PlasmidFinder/). ^*c*^ResFinder 3.2 (https://cge.cbs.dtu.dk/services/ResFinder/). ^*b*^ and ^*c*^ARGs and a replicon type that were identified on the same scaffold were defined as a plasmid that carried these ARGs. This was verified using BLAST-match of the ARGs-carrying scaffold with IncM2 pSEIL-3. ^*d*^[I] represents intermediate resistance phenotype. Antibiotics abbreviations: CTX-cefotaxime; AMC-amoxicillin/clavulanate; AMK-amikacin; GEN-gentamicin; TOB- tobramycin; TMS-trimethoprim-sulfamethoxazole; CIP- ciprofloxacin.*

### Local and Global Occurrences and Comparative Genomics of *S. enterica* Serovars Cerro, Havana, and Liverpool

In order to assess the origin of the *Salmonella enterica* serovars identified in this study, we analyzed the *Salmonella* national database that consists data on all human and non-human *Salmonella* isolates recovered in Israel (the reference *Salmonella* laboratory, the Ministry of Health, Israel). The data indicated that during the study period (2015–2017), the annual prevalence of Cerro, Havana and Liverpool serovars was relatively low; In human infections it ranged from 0.1–0.2% (out of an average of 3,952 *Salmonella* isolates/year). In non-human sources, the prevalence increased throughout these years but was also low (0, 0.2% and 1% for Cerro, Havana and Liverpool in 2015, to - 0.6%, 0.4%, and 3.3%, in 2017, respectively).

In order to evaluate the global abundance of these *Salmonella* serovars and to hypothesize about their main reservoirs we performed a meta-analysis on a global dataset of SRA *Salmonella enterica* isolates belonging to the respected serovars (Supplementary Table 2, *n* = 1394). The meta-analysis indicated that these serovars were recovered previously from various human, animal and food sources, with Cerro being the most prevalent serovar. This meta-analysis confirmed that these three serovars are reported herein for the first time in horses.

**FIGURE 2:**
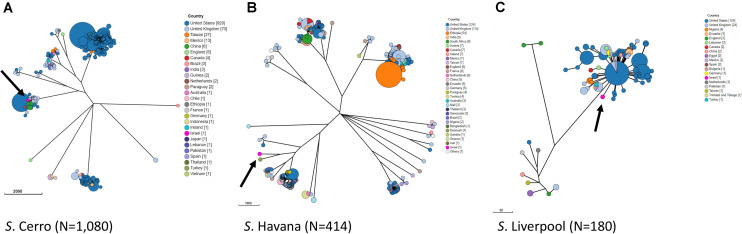
Phylogenetic analysis by wgMLST of global populations of *S*. Cerro **(A)**, *S*. Havana **(B),** and *S*. Liverpool **(C)**. GrapeTree visualization of a NINJA NJ tree based on wgMLST allelic distances. Strains with allelic distances of ≤10 alleles were clustered into a single node. The three Israeli strains of each serovar are shown in pink and are indicated by an arrow.

In order to study the relations with globally reported lineages and clusters, we compared our Israeli genomic sequences with all global genomes of *S*. Cerro, *S*. Havana, and *S*. Liverpool with geographical source, available in Enterobase database. A minimum spanning tree representing all wgMLST profiles for each serovar is shown in Figure 2. The analysis of the global population highlights closely clustered genotypes that originate from specific geographical locations. The Israeli genotypes did not significantly cluster with strains from other countries, and the minimum allelic distances from the nearest neighbors were 40 for *S*. Cerro, 779 for *S*. Havana, and 39 for *S*. Liverpool (Figure 2). The analysis indicated that the antibiotic resistance phenotypes of the Israeli strains are linked to genomic profiles unique to Israel.

### Characterization of the ESBL-Encoding *Salmonella* Plasmids

The WGS data revealed similar plasmid content between the serovars with a common IncM *bla*_*CTX–M–*__3_-encoding plasmid (Table 2). The *bla*_*CTX–M–*__3_-encoding plasmids of all twelve isolates were successfully transformed into *E. coli* DH10B The *bla*_*CTX–M–*__3__–_positive transformants possessed exactly the same antibiotic susceptibility profile showing resistance to all cephalosporins except for ceftazidime and co-resistance to trimethoprim/sulfamethoxazole and aminoglycosides (Supplementary Table 1).

**FIGURE 3:**
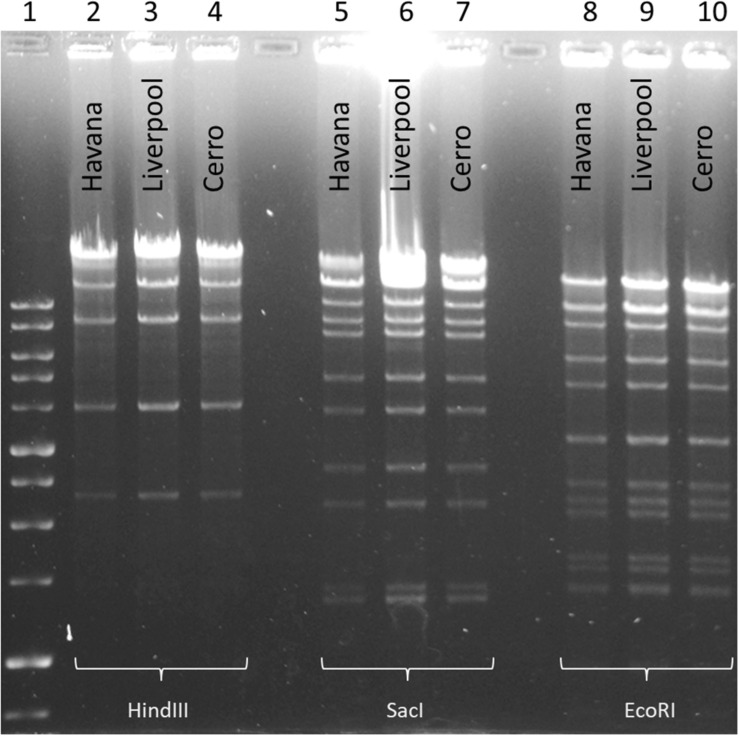
Plasmid RFLP mapping of the CTX-M-3-encoding plasmid DNA from *Salmonella* serovars. RFLP analysis of plasmid DNA (1 μg) isolated from three *Salmonella* isolates representing each of the serovars (*S.* Liverpool 667220; *S.* Havana 373.3.1; and *S.* Cerro 303.4.3) was restricted with three restriction enzymes, *Sac*I (Lanes 2–4), *Eco*RI (Lanes 5–7), and *Hin*dIII (Lanes 8–10). 1 kb DNA size ladder (Lane 1). Plasmid RFLP analysis revealed identical restriction patterns demonstrating the presence of an identical plasmid common to all the three serovars.

To examine and support the possible inter-serovar plasmid transmission we compared all 12 *bla*_*CTX–M–*__3_-encoding plasmids by plasmid-RFLP. Ten out of 12 plasmids (83%) showed an identical RFLP pattern, suggesting an inter-serovar horizontal plasmid transfer. We designated this plasmid as pSEIL-3 (plasmid of *S. enterica* from Israel encoding *bla*_*CTX–M–*__3_). *In silico* analysis of the WGS data confirmed the presence of pSEIL-3 in all three serovars. A representative RFLP analysis of pSEIL-3 is presented in Figure 3. Two out of the 12 *bla*_*CTX–M–*__3_-carrying ESBL-S isolates (229.2.2 and 347.2.2, Table 1) were IncM2 plasmids with a different RFLP pattern (Results are not presented) suggesting the presence of a variant of this plasmid (Table 1).

### Complete Sequence of pSEIL-3

**FIGURE 4:**
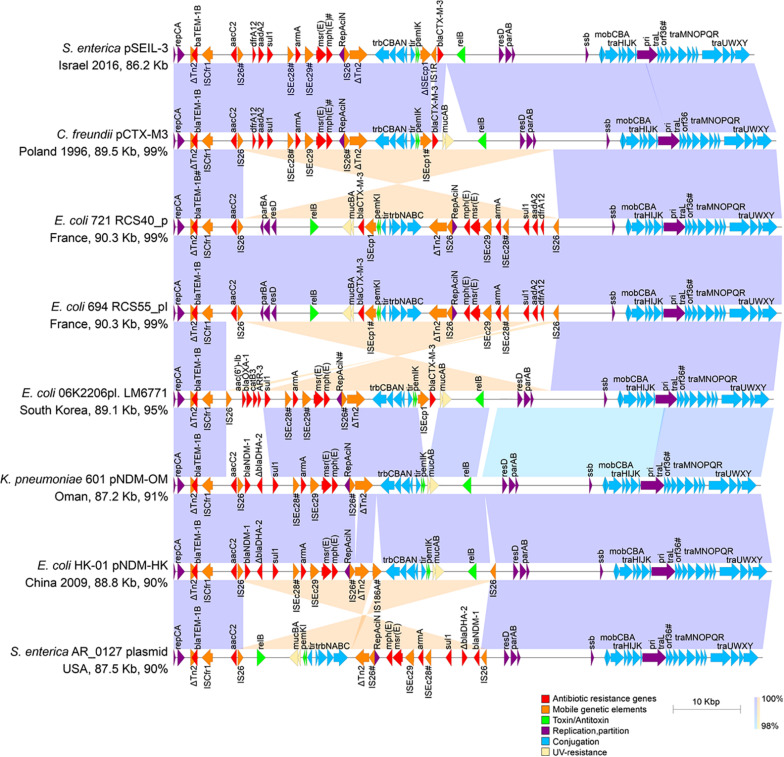
Linear plasmid maps of pSEIL-3 and seven highly related IncM2 related plasmids. Linear schematic maps of pSEIL-3 and IncM2-related plasmids retrieved from the GenBank (aligning for >90%). Plasmids’ GenBank accession numbers: pCTX-M3 - AF550415; RCS40_p - LT985241; RCS55_pI - LT985387; LM6771 - KX009507; pNDM-OM - JX988621; pNDM-HK - HQ451074; AR_0127 - CP032193. Arrows (colored by function) represent the major annotated gene groups and IS elements. The alignment between related plasmids is shown (purple, same direction alignment; orange, and opposite direction). The year of plasmid isolation is indicated when available. Truncated genes are labeled with ‘Δ’ and mutated genes (identity > 99%) are labeled with #.

To deepen our understanding on the transferability of pSEIL-3 we sequenced the purified plasmid (Havana 373.3.1) and performed long-read MinION sequencing. Using a hybrid assembly, we generated the complete sequence of the circular 86207-bp plasmid. pSEIL-3 was an IncM2 plasmid (Carattoli et al., 2015) and encoded 118 ORFs, 24 conjugation genes and a single toxin-antitoxin pair *pemIK*. The pSEIL-3 resistome encompassed nine ARGs conferring broad resistance to cephalosporins (*bla*_*TEM–*__1__*B*_ and *bla*_*CTX–M–*__3_), aminoglycosides (the modifying enzymes, *aac2* and *aadA2* and the 16S rRNA methyl transferase, *armA*), trimethoprim (*dfrA12*), sulfonamide (*sul1*) and to macrolides (*msrE* and *mphE*) (Figure 4).

Blast-based search of plasmids related to pSEIL-3 revealed that our sequenced plasmid was virtually identical to pCTX-M-3 from *Citrobacter freundii* except for a 3902 bp region that encodes *mucAB* of the *umuDC*-like gene family that is involved in UV-resistance, and four additional ORFs that encode hypothetical proteins in pCTX-M-3. The conjugation genes in both plasmids were identical except for a truncation in *orf36* that was shown previously to be involved in plasmid mobilization efficiency (Dmowski et al., 2018). In addition, pSEIL-3 resembled six other *bla*_*CTX–M–*__3_ and *bla*_*NDM*_-encoding plasmids aligning to >90% of its sequence (Figure 4). These plasmids were isolated from various human Enterobacterales strains (*E. coli* - 4; *C. freundii* - 1; *K. pneumoniae* - 1; *S. enterica* - 1) isolated from different countries and years, demonstrating the broad-host-range and high stability nature of these plasmids. Plasmid alignment revealed several DNA rearrangements that seemed to be host-dependent and presumably were linked to the presence of IS*26* (Figure 4).

### Transferability of pSEIL-3

Plasmid pSEIL-3 proved to be self-conjugable and was transferrable into both *E. coli* and *K. pneumoniae*. Acquisition of pSEIL-3 resulted in the same antibiogram as the donor ESBL-S strains (Supplementary Table 1).

In order to examine the *in situ* transferability of pSEIL-3 and pSEIL-3-like IncM2 plasmids in the horses’ gut, we screened 16 non-*Salmonella* isolates that co-colonized the horses, and that were PCR positive for *bla*_*CTX–M–*__1_ –group, for the presence of pSEIL-3 using a specific six-gene multiplex PCR we have developed (Table 3). We identified pSEIL-3 and pSEIL-3-like plasmids in 12/16 (75%) of the ESBL-E isolates tested. These isolates belonged to various Enterobacterales species, including *E. coli*, *K. pneumoniae, K. oxytoca*, and *C. freundii*. Therefore, we defined it as a broad host range plasmid (Figure 1).

**TABLE 3:** Description of the genes and primers used for the screening of pSEIL-3 multiplex PCR.

**Gene**	**Primer ID**	**Sequence**	**Product size**	**Primers coordinates on pSEIL-3 (5′-3′)**	
replicon	IncM2_FW	GGATGAAAACTATCAGCATCTGAAG	786	86138	86162
	IncM2_RV	CTGCAGGGGCGATTCTTTAGG		716	696
aminoglycoside resistance	armA_F	GGGGTCTTACTATTCTGCCTAT	521	18264	18285
	armA_R	GCTGGTAATTCTCTTCCATTCC		18784	18763
*bla*CTX-M-1 ESBL	CTX-M1-F	AAAAATCACTGCGCCAGTTC	415	39216	39235
	CTX-M1-R	AGCTTATTCATCGCCACGTT		39630	39611
pSEIL-3 backbone region	pSEIL3_orf_korC_F	CTGGGACCGGATGCGTGAT	1315	53404	53422
	pSEIL3_orf_korC_R	TCGTTTTGATGTTGCGCCGG		54718	54699
Tra	traJ_F	CGGACTGATATGCGGCGAGA	267	67834	67853
	traJ_R	AGGCGGTTAAGGAGCTCACC		68100	68081
Tra	pSEIL3_traUX_F	TGCGATCCTGGACATGCAAAAC	997	80710	80731
	pSEIL3_traUX_R	TGTTAATCAGCGTGGCCTGGAT		81706	81685

*The multiplex PCR was performed with PCRBIO HS Taq Mix Red (PCRBIO-systems, United Kingdom) at the following conditions: denaturation at 95°C for one minute, 29 cycles of denaturation (95°C, 15 s), annealing (61.1°C, 15 s), and elongation (72°C, 90 s).*

## Discussion

In this study, we report for the first time the emergence of three MDR CTX-M-3-producing *S. enterica* serovars - Cerro, Havana and Liverpool, which colonize and cause severe infections in hospitalized horses. Based on WGS and molecular studies we elucidated the route of ESBL spread in *S. enterica* and discovered an inter-serovar horizontal transfer of an IncM2 broad host range plasmid, pSEIL-3. Furthermore, we identified the clonal expansion of *bla*_*CTX–M–*__3_-producing *S.* Cerro that was responsible for more than half of the cases.

Global phylogenetic serovar analysis indicated the genetic uniqueness of our strains, and the metadata analysis revealed that these three serovars have not been described before in horses. Previously, *S.* Cerro was mainly reported in cattle in the United States (Tewari et al., 2012; Webb et al., 2017), and as the main causative Salmonellosis pathogen in dairy farms (Van Kessel et al., 2007; Kovac et al., 2017). In the United States and the Far East, *S*. Cerro has also been reported in poultry (Roy et al., 2002; Murase et al., 2004). The second serovar we found, *S*. Havana was reported both in humans (Backer, 2000; Bekal et al., 2013) and in poultry (Clemente et al., 2013), and less frequently in wild birds (Reche et al., 2003) and in environmental setting (Magwedere et al., 2015). It was also identified previously as an ESBL-producer, carrying various *bla*_*CTX–M*_ alleles (Bekal et al., 2013; Clemente et al., 2013). *S*. Liverpool is a more rarely reported serovar, with a single report that describes its origin from cattle feces in an EU registered slaughterhouse (Madden et al., 2007).

The source of the *S. enterica* serovars that we identified is unknown. In spite a large nation-wide survey of poultry-associated *Salmonella enterica* was recently reported from Israel (Cohen et al., 2020), data on the serovars that are circulating in the community or in hospital equine populations is still lacking. The large animal department in the KSVM-VTH serves equine patients from diverse farms that occasionally may be housed together with different farm animals. In addition, various animals, often rescued from rural areas, are sporadically admitted for intensive care to the same department. These farm animals may be the source for these *Salmonella* serovars however, a solid support for this is lacking.

Interestingly, the majority of the horses included in our study were not detected as positive ESBL carriers on admission to the hospital, suggesting the nosocomial acquisition of the ESBL-producing strains or the ESBL genetic elements (the *bla*_*CTX–M–*__3_ gene or its encoding plasmid). In the United States, studies that describe asymptomatic community carriage of *Salmonella* in horses report the prevalence of 0.8% without information on the existing serovars (Traub-Dargatz et al., 2000). Other studies in horses that describe the prevalence of clinical *Salmonella* isolates indicate that the main serovars are Typhimurium, Newport, Agona, Javiana, Anatum, Infantis, and Braenderup (Hernandez et al., 2014; Martelli et al., 2019). Nevertheless, the serovars that we describe herein are unique and are mentioned for the first time in the context of equine population.

Dissemination of ESBL among the hospitalized horses showed a complex epidemiology that included the clonal expansion of *S*. Cerro between seven horses alongside with an in-hospital spread of pSEIL-3 that horizontally transferred to all three *Salmonella* serovars. Acquisition of this single plasmid with its wide resistome was responsible for the dissemination of multidrug resistance. Complete plasmid sequencing of pSEIL-3 indicated that it is merely identical to the previously reported wide-host-range pCTX-M-3 plasmid from *Citrobacter freundii* (Golebiewski et al., 2007) and to other MDR plasmids, that encode various carbapenemases, all from human origin. The findings of pSEIL-3-like IncM2 plasmids in other non-*Salmonella* ESBL-E species that colonized the horses’ gut is alarming, and proves their high inter-species transmissibility. The presence of pSEIL-3 in horses, and previously in humans, highlights the risk of horizontal transmission of MDR plasmids between human, animals and environmental pools.

The potential transmission of pSEIL-3-like plasmids is disturbing not only due to their broad host range, but also due to their wide resistome, which confers resistance to all aminoglycosides and to trimethoprim/sulfamethoxazole. Considering the massive use of aminoglycosides antibiotics, often combined with β-lactamase inhibitors, for treating ESBL-producing pathogens in humans, food and companion animals, emphasizes the risk of this plasmid as it may lead to limited treatment options. Additional reports regarding this clinically important ARGs combination in *S. enterica* are infrequent, with one recent study that described a similar MDR pattern of *S. Virchow* from food animals in South Korea (Na et al., 2020), and another study describing shedding of quinolone resistant and ESBL-producing *S. enterica* serovars in swine population in the United States (Elnekave et al., 2019).

The clinical impact of ESBL-S and specifically pSEIL-3-like plasmids in a ‘One-Health’ perspective is vast. The clonal expansion of the *S*. Cerro underlines the lack of current infection-control measures for detecting and controlling *Salmonella* infections in the veterinary hospital, and calls for the implementation of control measures to prevent further spread. The existence of highly transmissible plasmids such as pSEIL-3 and its spread into three uncommon *S. enterica* serovars highlights the importance of detailed molecular analyses for elucidation of these transmission paths. The developed multiplex PCR in this study enables the tracking of pSEIL-3 in future studies and in active surveillance actions.

This study describes horse-to-horse spread of a zoonotic pathogen harboring a wide-host-range MDR plasmid, which was reported previously in human pathogens, representing a major public health concern. Although the source of this highly transferable plasmid in the veterinary hospital and its circulating routes remains unclear, its disseminative nature is alarming.

## Data Availability Statement

The datasets presented in this study can be found in online repositories. The names of the repository/repositories and accession number(s) can be found below: https://www.ncbi.nlm.nih.gov/genbank/, SAMN12532153; https://www.ncbi.nlm.nih.gov/genbank/, SAMN12532154; https://www.ncbi.nlm.nih.gov/genbank/, SAMN12532152.

## Author Contributions

ZD and AS-T collected the specimens. ZD performed all the microbiological and molecular analyses. KK assisted in the bioinformatics analysis. MD-C and AR performed the serovar typing, the PFGE and the wgMLST analyses. AS was involved in the study design. SN-V was responsible for the design of the study and data analyses. ZD and SN-V wrote the manuscript. All authors read and approved the submitted manuscript.

## Conflict of Interest

The authors declare that the research was conducted in the absence of any commercial or financial relationships that could be construed as a potential conflict of interest.
