# Supplementary material for: Emergence and Spread of Different ESBL-Producing Salmonella enterica Serovars in Hospitalized Horses Sharing a Highly Transferable IncM2 CTX-M-3-Encoding Plasmid
Source: Front Microbiol. 2020 Dec 17;11:616032. doi: 10.3389/fmicb.2020.616032 (PMC7773750; doi:10.3389/fmicb.2020.616032)
Supplement: Supplementary file 1 [file Data_Sheet_1.docx]

**Supplementary data**

**Table S1.** Antibiotic susceptibility profile of pSEIL-3-carrying *Salmonella* isolate 373.3.1 and its *E. coli* J53 transconjugant

| Drug^a^ | MIC (mg/L)^b^ | | |
| --- | --- | --- | --- |
|  | *Salmonella enterica serovar* Havana  strain 373.3.1 | *E. coli* J53RifR recipient strain | *E. coli* J53[pSEIL-3] |
| AMP | ≥32 | ≤2 | ≥32 |
| CRO | ≥64 | 4 | ≥64 |
| CTX | ≥64 | ≤1 | ≥64 |
| CAZ | 4 | ≤1 | ≥64 |
| ERT | ≤0.5 | ≤0.5 | ≤0.5 |
| MER | ≤0.25 | ≤0.25 | ≤0.25 |
| IMP | ≤0.25 | ≤0.25 | ≤0.25 |
| AMC | 16 | ≤2 | 4 |
| TZP | ≤4 | ≤4 | ≤4 |
| AMK | ≥64 | 4 | ≥64 |
| GEN | ≥16 | ≤1 | ≥16 |
| CIP | ≤0.25 | ≤0.25 | ≤0.25 |
| OFX | ≤0.25 | ≤0.25 | ≤0.25 |
| TMS | ≥320 | ≤20 | ≥320 |
| FOS | ≤16 | ≤16 | ≤16 |
| NIT | ≤16 | ≤16 | ≤16 |
| ARGs^c^ | *bla*_TEM-1_, *bla*_CTX-M-3_, *armA*, *aadA2*, *aac(3)IIb*, *dfrA12* |  | *bla*_TEM-1_, *bla*_CTX-M-3_, *armA*, *aadA2*, *aac(3)IIb*, *dfrA12* |

**^a^** Antibiotics abbreviations: AMP - ampicillin; CRO - ceftriaxone; CTX - cefotaxime; CAZ - ceftazidime; ERT - ertapenem; MER - meropenem; IMP - imipenem; AMC- amoxicillin/clavulanate; TZP - piperacillin-tazobactam; AMK - amikacin; GEN -gentamicin; CIP - ciprofloxacin; OFX - ofloxacin; TMS - trimethoprim/sulfamethoxazole; FOS - fosfomycin; NIT - nitrofurantoin.

**^b^** MIC interpretation: Intermediate - light grey; Resistant - dark grey.

**^c^** Antibiotic resistance genes (ARGs) encoded by pSEIL-3 which explain the resistance phenotype, in the donor and in the recipient strains.

**Table S2.** SRA meta-analysis of *Salmonella enterica* serovars identified in this study and their isolation sources

| *Salmonella enterica* Serovar | No. of SRA isolates | No. of isolates of the respective serovar according to the isolation source ^a^  (Phi coefficient) ^b^ | | | | | | | | |
| --- | --- | --- | --- | --- | --- | --- | --- | --- | --- | --- |
|  |  | human | equine | cattle | swine | avian | animal | food | environment | UD |
| Cerro | 1032 | 67  (-0.38) | 0 | 732 (0.54) | 68 | 21 (-0.13) | 16 (-0.13) | 75 (-0.19) | 16 | 37 |
| Havana | 253 | 113 (0.4) | 0 | 14 (-0.47) | 15 | 8 | 12 | 58 (0.19) | 6 | 27 |
| Liverpool | 109 | 22 | 0 | 19 (-0.22) | 15 | 19 (0.22) | 11 (0.13) | 16 | 0 | 7 |
| **Total** | 1394 | 202 | 0 | 765 | 98 | 48 | 39 | 149 | 22 | 71 |

^a^ The isolation source (eight categories) was identified based on SRA metadata mining using the following keywords: **'human'** - 'human', 'homo sapiens'; **'equine'** - 'horse', 'equine', 'foal'; **'cattle'** - 'cattle', 'bos taurus', 'bovine', 'dairy', 'beef', 'raw milk'; **'swine'** - 'pig', 'swine', 'pork', 'market hog'; **'avian'** - 'avian', 'chicken', 'turkey', 'duck', 'bird'; **'animal'** - 'animal', 'dog', 'cat', 'felis catus', 'canis lupus', 'sus scrofa', 'oyster', 'shrimp', 'reptile'; **'food'** - 'food'; **'environment'** - 'environment', ‘water', 'soil', 'bone meal'.

**^b^** Phi coefficient values represent the correlations between a specific serovar and the isolation source supported by p-values <0.01 (step-down method multiple test correction using Bonferroni adjustments, alpha = 0.01).

UD – an undefined source.

**
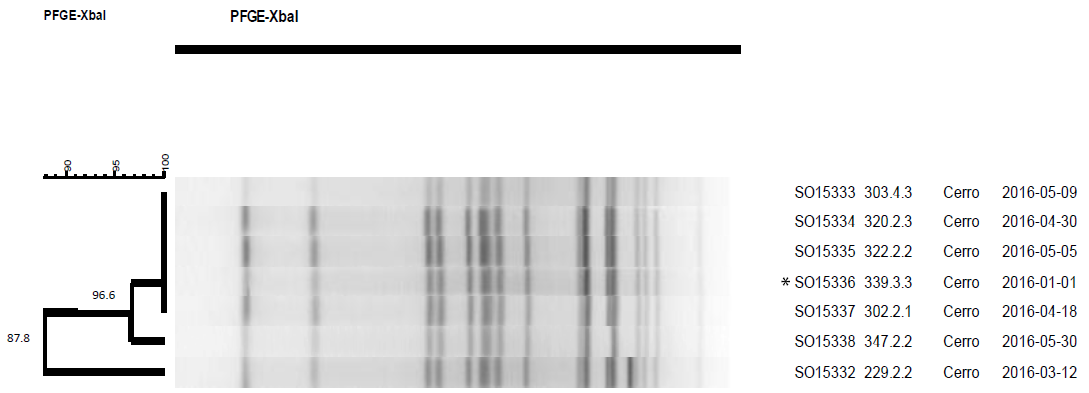
**

**Figure S1. PFGE of seven *Salmonella* *enterica* Cerro isolates recovered from hospitalized horses.** Seven isolates were analyzed using PFGE following *XbaI* restriction. The PFGE pulsotype of five isolates was identical (339.3.3, 303.4.3, 320.2.3, 322.2.2, 302.2.1). Two isolates (347.2.2, 229.2.2) exhibited a single band difference resulting in 96.6% and 87.8% similarity with the dominant pulsotype. Asterisk (*) marks the Illumina sequenced isolate.
